# Trends in active surveillance for very low‐risk prostate cancer: do guidelines influence modern practice?

**DOI:** 10.1002/cam4.1132

**Published:** 2017-09-18

**Authors:** Rahul R. Parikh, Sinae Kim, Mark N. Stein, Bruce G. Haffty, Isaac Y. Kim, Sharad Goyal

**Affiliations:** ^1^ Department of Radiation Oncology Rutgers Cancer Institute of New Jersey New Brunswick NJ 08903; ^2^ Biometrics Division Rutgers Cancer Institute of New Jersey New Brunswick NJ 08903; ^3^ Department of Biostatistics Rutgers School of Public Health New Brunswick NJ 08903; ^4^ Department of Medical Oncology Rutgers Cancer Institute of New Jersey New Brunswick NJ 08903; ^5^ Department of Urology Rutgers Cancer Institute of New Jersey New Brunswick NJ 08903

**Keywords:** Active surveillance, disparities, guidelines, national cancer database, prostate cancer

## Abstract

As recommended by current NCCN guidelines, patients with very low‐risk prostate cancer may be treated with active surveillance (AS), but this may be underutilized. Using the National Cancer Database (NCDB), we identified men (2010–2013) with biopsy‐proven, very low‐risk prostate cancer that met AS criteria as suggested by Epstein (stage ≤ T1c; Gleason score (GS) ≤ 6; PSA < 10; and ≤2 [or <33%] positive biopsy cores) and aged ≤76, and low comorbidity index (Charlson‐Deyo score = 0). For those patients meeting this criteria, we performed generalized estimation equation (GEE) method with incorporation of correlation in patients clustered within facility to determine the likelihood of undergoing AS. Among the 448 773 patients in the NCDB with low‐risk prostate cancer, 40 839 patients met the inclusion criteria. AS was utilized in 5798 patients (14.2%), while within the very low‐risk patients receiving treatment, up to 52.2% received radical prostatectomy. In univariate analyses, AS utilization was associated with older age, uninsured status (compared to private insurance), farther distance from facility, academic/research institutions and particularly in the New England region (all *P* < 0.01). After adjustments of other predictors in multivariate analysis, patients preferentially received AS if they were older (all OR's > 1 compared to younger groups), uninsured (vs. any insurance type, OR's > 1); or treated at academic/research center (OR > 1). The overall use of AS increased from 11.6% (2010) to 27.3% (2013). We found a low, but rising rate of AS in a nationally representative group of very low‐risk prostate cancer patients. Disparities in the use of AS may be targeted to improve adherence to national guidelines.

## Introduction

Prostate cancer is the most common cancer in men, with approximately 233 000 patients being diagnosed each year 1. Patients with localized, favorable/low‐risk prostate cancer represent the majority of these diagnoses, and are eligible for a myriad of treatment paradigms, including radical prostatectomy (RP), external‐beam radiation therapy (RT), brachytherapy (BT), androgen deprivation therapy (ADT), and active surveillance (AS).

AS is based on the premise that a patient population exists that may not benefit from primary treatment of their prostate cancer and has two goals: (1) to provide definitive treatment for men with localized cancers that are likely to progress and (2) to reduce the risk of treatment‐related complications for men with cancers that are not likely to progress. Conceptually, this form of treatment was developed due to concerns about both over‐diagnosis and over‐treatment of prostate cancer given that patients diagnosed with prostate cancer are more likely to die of nonprostate cancer causes and may be unnecessarily exposed to treatment‐related morbidity with limited long‐term survival advantage 2. In fact, two board specialty societies have recently developed “choosing wisely” campaigns focused on PSA screening and AS 3, 4.

Multiple prospective studies, which total less than 3000 patients, have evaluated the experience of treating patients with AS, using their respective eligibility criteria 5, 6, 7, 8, 9, 10. Long‐term follow‐up (median follow‐up of 6.4 years, range 0.2–19.8 years) of the University of Toronto series (*n* = 993), by Klotz et al. 11 revealed the safety and feasibility of AS given that only 2.8% of patients developed metastatic disease and only 1.5% of patients died from prostate cancer. In 2010, the NCCN guidelines first introduced recommendations to incorporate AS in clinical practice 12, which reflected criteria developed by Epstein 13, 14, D'Amico 15, and Klotz 11. More than 20 years ago, Epstein et al. developed specific criteria using serum PSA level, PSA density, and needle biopsy pathologic findings to accurately predict (up to 90% of cases) “insignificant prostate cancer” that may undergo AS 16. This collection of criteria was later modified with no limited difference in altering the detection of non‐organ confined prostate cancer 17. Thus, the current Epstein criteria provides excellent accuracy, even in the modern era of extended biopsy sampling 18, and provides an excellent 15‐year prostate‐cancer‐mortality of only 0.4% 13.

Recently published data by the Prostate Testing for Cancer and Treatment (ProtecT) study group revealed no difference in prostate cancer‐specific mortality irrespective of AS or active intervention 19. Currently, there is limited clinical data evaluating contemporary, nationwide trends for the utilization of AS for patients with very low‐risk prostate cancer in the United States following the 2010 NCCN recommendations. To address these issues, we used a representative cohort of very low‐risk prostate cancer patients from the National Cancer Database(NCDB), to examine trends and disparities in adherence to appropriate national guideline recommendations for AS.

## Patients and Methods

### Data source

The NCDB, a national hospital‐based oncology database, was used to conduct a retrospective, cohort study of patients with verylow‐risk prostate cancer diagnosed from 2010 to 2013. This was a time period [2010 onwards] after which AS and the term “very low risk” prostate cancer was first incorporated into national guidelines 12 and was coded within the NCDB as a hospital reporting standard for the American College of Surgeons(ACS) and Commission on Cancer (CoC). As a joint project of the ACS/CoC and the American Cancer Society, the NCDB is a prospectively collected registry from 1500 hospitals representing approximately 70% of all cancers diagnosed in the US with accumulated data on 29‐million cancer cases.

### Study patients

**Figure 1:**
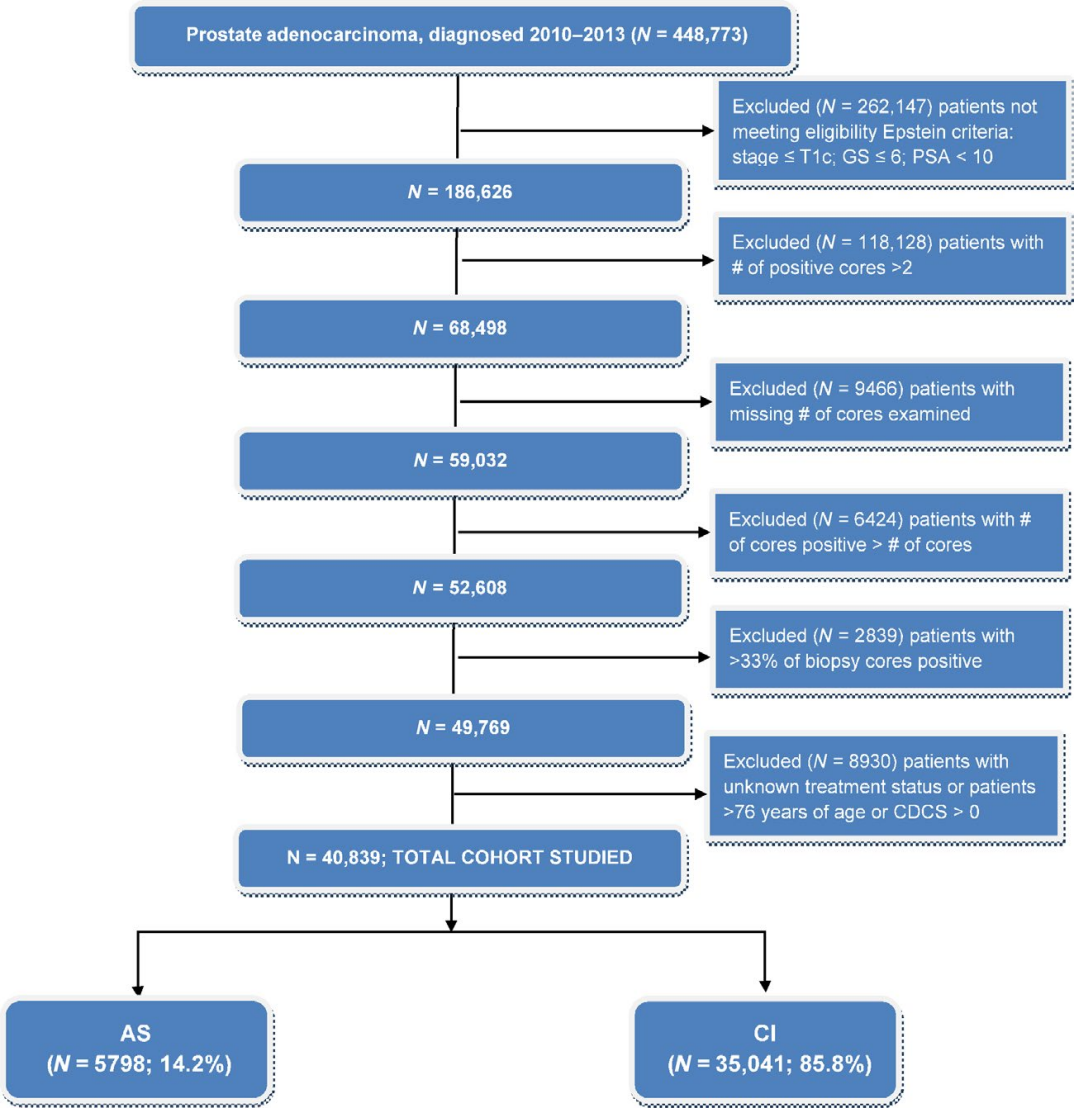
CONSORT diagram. AS, active surveillance; CI, curative intervention; GS, Gleason score.

The CONSORT diagram in Figure 1 shows the study exclusion criteria used to define the cohort. Of the 1 208 180 patients diagnosed with prostate cancer from 2004 to 2013, there were 448 773 patients available in 2010–2013. Patients under the Epstein criteria (stage ≤ T1c; Gleason score ≤ 6; PSA < 10; and positive biopsy cores <33% (or the number of positive cores ≤2) are 52 608. Patients with no treatment or unknown treatment status were removed, resulting in, 49 769. We also excluded patients who were older than 76 years old or Charlson Deyo score > 0. Further, we excluded facility with only one patient before statistical analyses for correlated binary responses using generalized estimating equations (GEE) were applied. This left 40 839 patients from 1155 facilities for analyses of association of AS and a set of factors.

### Outcome and variables

The primary outcome of interest is whether patients in very low‐risk group received treatment or opted for AS. Among patients who were treated, different types of primary interventions were summarized as follows: RP, ADT, external‐beam RT, or BT. To analyze the patterns in AS, we investigated patients’ demographic/socio‐economic characteristics such as age, race, insurance status, income and education and also facility information like facility type, location or hospital volume. Patients’ age was categorized into two groups (≤61 years and 62–76 years) and race into four groups (white, black, others and unknown). Median household income was classified into four levels (≤$38 000; 38 000–47 999; 48 000–62 999; and ≥63 000) and education based on percent of no‐high school degree in patient's zip code was coded in the following four categories (≥21%; 13–20%; 7–12.9%; and <7%). There were 1155 unique hospitals available in data and four different types of facilities – facilities – community cancer center, comprehensive cancer center, Academic/research hospital, or integrated facility. These hospitals were located in nine different regions, “New England”; “Middle Atlantic”; “South Atlantic”; “East‐North central”; “East‐South central”; “West‐North Central”; “West‐South Central”; “Mountain”, or “Pacific”. To define hospital volume, the median number of surgical procedures per facility per year in 2010–2013 was calculated. “High” hospital volume was designated for a facility if its median number of surgical procedures was greater than the 75th percentile of the median number of cases (>59 cases/year).

### Statistical analysis

Patients’ baseline characteristics and facility information were summarized using counts and proportion for treatment and AS group. The generalized estimating equations (GEE) approach was employed to analyze the association of AS status clustered within facility with predictors of interest, in univariate setting and also in multivariable model. For each of the models, the logit link function was employed to relate probability of undergoing AS and a set of covariates and exchangeable correlation structure within facility was assumed. Variables whose *P*‐value for a significance test is less than 0.3 in univariate setting were selected to build up the multivariable model. Unadjusted and adjusted odds ratio (OR) of undergoing AS were reported for each of variables in the models along with 95% confidence intervals. All statistical tests were performed in two‐sided at a significance level 0.05, and *P*‐values were provided. Statistical analyses were performed using SAS 9.4 (Cary, NC).

## Results

### Descriptive statistics

**Figure 2:**
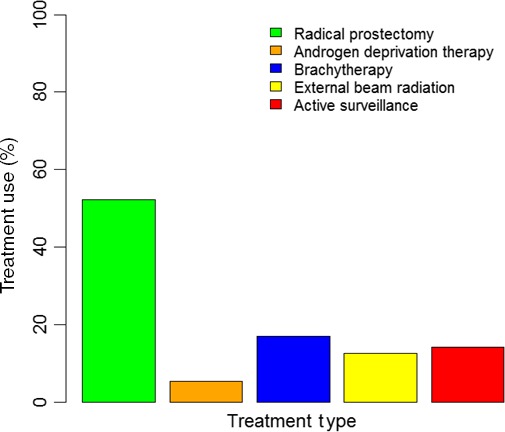
Use of active surveillance and various curative intervention modalities in very low‐risk prostate cancer patients. Note that treatment interventions are not mutually exclusive; patients may have received multiple treatment modalities.

There were 448 773 patients who were diagnosed prostate cancer in 2010–2013 available for analyses. Very low‐risk prostate cancer patients by the Epstein criteria were 40 838. Among them, 5798 (14.2%) patients were prescribed AS (Figure 2). Within the eligible cohort, there was an increase in use of AS from 11.6% in 2010 to 27.3% in 2013. Among patients who opted for treatment, 21 311 (52.2%) patients underwent RP (Figure 2). More patients with the longer life expectancy (≤61 years) received curative intervention compared to older patients (88.1% vs. 83.9%). White patients were of majority of the population (>80%). Patients without insurance were more likely to received AS compared to patients with insurance (22.1% vs. all other insurance types <16%). Patients who were diagnosed at comprehensive or integrative cancer centers were more likely to receive curative treatments compared to community cancer programs or academic/research facilities (>90% vs. <86%) (Table 1).

**Table 1:** Patient population characteristics of cohort eligible for AS by Epstein criteria

		Curative intervention	Active surveillance		
		*N* = 35 040	*N* = 5798		
Age	Mean (SD)	61.8 (7.2)	63.3 (7)	<0.0001	Two sample t‐test
	Median (min–max)	62 (28–76)	64 (28–76)	<0.0001	Wilcoxson
Age group, *N* (%)	≤61 years old	16 249 (88.1)	2190 (11.9)	<0.0001	
	62–76 years old	18 791 (83.9)	3608 (16.1)		
Race, *N* (%)	White	29 157 (85.8)	4830 (14.2)	<0.0001	
	Black	4463 (87.5)	636 (12.5)		
	Others	924 (80.6)	223 (19.4)		
	Unknown	493 (82)	109 (18)		
Insurance status, *N* (%)	Not insured	434 (77.9)	123 (22.1)	<0.0001	
	Private	21 170 (87)	3150 (13)		
	Medicaid	595 (85.7)	99 (14.3)		
	Medicare	11 732 (84.1)	2217 (15.9)		
	Other government	667 (89.2)	81 (1.4)		
	Unknown	442 (77.5)	128 (22.5)		
Facility type, *N* (%)	Community cancer program	2315 (85.8)	384 (14.2)	<0.0001	
	CCC	15 231 (91.1)	1490 (8.9)		
	Academic/research	14 845 (80.1)	3688 (19.9)		
	Integrated	2649 (91.8)	236 (8.2)		
Facility location, *N* (%)	New England	2165 (77.2)	639 (22.8)	<0.0001	
	Middle Atlantic	5560 (81.2)	1289 (18.8)		
	South Atlantic	8212 (89)	1015 (11)		
	East North Central	6330 (86)	1029 (14)		
	East South Central	3050 (92.4)	252 (7.6)		
	West North Central	3432 (87.3)	500 (12.7)		
	West South Central	1847 (91.2)	178 (8.8)		
	Mountain	1134 (87.2)	167 (12.8)		
	Pacific	3310 (82)	729 (18)		
Hospital volume, *N* (%)	High	23 013 (84.9)	4090 (15.1)	<0.0001	
	Low	12 027 (87.6)	1708 (12.4)		
Median income quartiles (2008–2012), *N* (%)	<$38 000	4772 (87.4)	691 (12.6)	<0.0001	
Missing (*N*) = 183	$38 000–$47 999	7239 (87.2)	1065 (12.8)		
	$48 000–$62 999	9310 (86.4)	1470 (13.6)		
	$63 000+	13 567 (84.2)	2541 (15.8)		
No high school degree (2008–2012), *N* (%)	>=21%	4360 (87.5)	625 (12.5)	<0.0001	
Missing (*N*) = 164	13–20%	8057 (87.3)	1168 (12.7)		
	7.0–12.9%	11 548 (85.8)	1919 (14.2)		
	<7%	10 938 (84.2)	2059 (15.8)		
Distance	0–5.6 miles	8705 (85)	1540 (15)	0.016	
Missing (*N*) = 145	5.7–12.6 miles	8815 (86.4)	1391 (13.6)		
	12.7–29.6 miles	8631 (85.6)	1448 (14.4)		
	>29.6 miles	8764 (86.2)	1399 (13.8)		

### Univariate analyses

Age, insurance type, distance to facility along with facility type and location are statistically significantly associated with AS in the univariate setting. Older patients (62–76 years) tended to be in AS group compared to patients with longer life expectancy (≤61 years, OR = 1.59, 95% CI = [1.39, 1.81]; Table 2). Patients who did not have insurance were more likely to receive AS compared to patients with private insurance (OR=1.75; 95% CI = [1.28, 3.1]). Patients who lived further away from the medical facilities were less likely to get AS compared to the group of patients who lived closer (OR = 0.81; 95% CI = [0.71, 0.91]; OR = 0.55; 95% CI = [0.44, 0.68]). Patients who were diagnosed in comprehensive cancer center was less likely to receive AS compared to patients in academic/research facilities (OR = 0.48; 95% CI = [0.38, 0.6]). Facilities located in New England were more likely to suggest AS to patients compared to all other areas (Table 2).

**Table 2:** Univariate and multivariate logistic regression analysis: association of AS status with variables of interest

	Group	Univariate analysis		Multivariate analysis	
		OR (95% CI)	*P*‐value	OR (95% CI)	*P*‐value
Age (ref = “≤61 years”)	62–76 years	1.59 (1.39, 1.81)	<0.0001	1.04 (1.02, 1.05)	<0.0001
Race (ref = “Black”)	White	1.03 (0.89, 1.19)	0.699	Not included	
	Others	1.27 (0.95, 1.69)	0.111		
	Unknown	1.13 (0.72, 1.78)	0.587		
Insurance status (ref = “Private”)	None	1.75 (1.28, 3.1)	0.0004	1.07 (1.03, 1.11)	0.0003
	Medicaid	1.00 (0.69, 1.44)	0.989	0.99 (0.96, 1.02)	0.46
	Medicare	1.53 (1.37, 1.7)	<0.0001	1.02 (1.01, 1.03)	<0.0001
	Other government	0.87 (0.62, 1.22)	0.425	0.99 (0.96, 1.01)	0.247
	Unknown	1.89 (1.45, 2.47)	<0.0001	1.07 (1.04, 1.11)	<0.0001
Facility type (ref = “Academic/research”)	Community cancer program	0.85 (0.64, 1.12)	0.252	0.96 (0.93, 0.995)	0.025
	CCC	0.48 (0.38, 0.6)	<0.0001	0.92 (0.90, 0.95)	<0.0001
	Integrated	0.64 (0.39, 1.06)	0.082	0.95 (0.91, 0.999)	0.047
Facility location (ref = “New England”)	Middle Atlantic	0.45 (0.30, 0.65)	<0.0001	0.91 (0.86, 0.95)	<0.0001
	South Atlantic	0.37 (0.25, 0.54)	<0.0001	0.90 (0.86, 0.95)	<0.0001
	East North Central	0.53 (0.37, 0.76)	0.001	0.92 (0.88, 0.97)	0.0002
	East South Central	0.34 (0.20, 0.58)	<0.0001	0.91 (0.86, 0.96)	0.0003
	West North Central	0.49 (0.31, 0.79)	0.003	0.94 (0.88, 0.99)	0.019
	West South Central	0.26 (0.16, 0.43)	<0.0001	0.89 (0.85, 0.93)	<0.0001
	Mountain	0.33 (0.17, 0.65)	0.001	0.91 (0.85, 0.97)	0.002
	Pacific	0.62 (0.4, 0.93)	0.023	0.95 (0.90, 1.01)	0.085
Hospital volume (ref = “low”)	High	0.96 (0.77, 1.19)	0.69	Not included	
Median income quartiles (2008–2012) (ref = “<$38 000”)	$38 000–$47 999	0.93 (0.82, 1.05)	0.22	Not included	
	$48 000–$62 999	0.99 (0.87, 1.11)	0.818		
	$63 000+	1.05 (0.92, 1.19)	0.486		
No high school degree (2008–2012) (ref = “>=21%”)	13–20%	0.98 (0.87, 1.11)	0.791	Not included	
	7.0–12.9%	1.02 (0.91, 1.15)	0.736		
	<7%	1.09 (0.96, 1.24)	0.203		
Distance (ref = “0–5.6 miles”)	5.7–12.6 miles	0.93 (0.85, 1.02)	0.109	0.99 (0.98, 1.00)	0.131
	12.7–29.6 miles	0.81 (0.71, 0.91)	0.001	0.98 (0.97, 0.99)	0.0005
	>29.6 miles	0.55 (0.44, 0.68)	<0.0001	0.94 (0.92, 0.95)	<0.0001

### Multivariable analyses

Variables whose *P*‐values from a significance test in univariate setting were <0.3 were selected to build the multivariable model. They include age, insurance status, distance from facilities, facility type and location. Similarly in univariate setting, older patients or patients without insurance were more likely to receive AS compared to the younger group or patients with private insurance group, respectively (OR=1.04; 95% CI = [1.02, 1.05]; OR = 1.07; 95% CI = [1.03, 1.11]). Compared to academic/research facilities, all others are less likely to recommend AS to their patients (ORs <1; *P*‐values <0.05). Patients who live further from the facilities were less likely to receive AS compared to the ones resided closer to the facilities (Table 2). Facilities located in New England were more likely to implement AS in very low‐risk patients compared to all others.

### Sensitivity analysis

To address any potential misclassification and underrepresentation of patients receiving AS, a sensitivity analysis was performed to include patients designated as receiving “no treatment.” This led to an additional 1996 patients for a total of 42 834 patients in the sensitivity analysis. As per yearly trends, when including patients receiving “no treatment” and AS, we found the rates of “no treatment” were 13.1%, 16.5%, 20.4%, and 24.4%, for 2010, 2011, 2012, and 2013, respectively. Thus, in this sensitivity analysis, the use of AS and/or “no treatment” increased from 13.1% in 2010 to 24.4% in 2013.

## Discussion

In this study, we found low rates of utilization of AS in a conservatively‐defined cohort of very low‐risk prostate cancer patients. With concerns of over‐diagnosis and over‐treatment of prostate cancer, the current national guidelines suggest that patients who meet appropriate criteria should undergo AS with conversion to curative treatment at evidence of progression (NCCN Guidelines V 2.2017). Recent ASCO (American Society of Clinical Oncology) clinical practice guidelines have endorsed Cancer Care Ontario's guidelines on AS 20, concluding that select patients with low volume, low‐intermediate risk prostate cancer may be offered AS. Several factors may also be taken into account for this recommended disease management strategy including age, prostate cancer volume, patient preference, and ethnicity.

As seen in this study, it is clear that a strict criteria used for AS (Epstein criteria), leads to increased use, as providers may be less likely to miss clinically relevant disease. We have identified specific patient and clinical disparities in the use of AS, particularly age, insurance status, distance from and type of treatment facility. In our study, even after adjusting for comorbidity index and clustered facilities in the generalized estimation equation method (with incorporation of correlation in patients clustered within facility to adjust for provider/institutional preference) in our multivariable model, the likelihood of patients over the age of 62 undergoing AS was almost 5% more those patients less than the age of 62 (OR = 1.04, multivariate model) and uninsured patients were less likely to undergo curative treatment rather than AS. Our findings may also reflect adherence to current national recommendations regarding stratification by age for prescribing surveillance regimens 21 and provider comfort with AS for patients within an older age range (perhaps with competing causes of death).

As a contemporary cohort in the literature, representing >70% of nationwide diagnoses of patients with very low‐risk prostate cancer, treatment at a comprehensive cancer center (where sophisticated treatment tools may exist) was more likely associated with curative intervention (compared to AS) than treatment at academic/research institution. Of course, AS programs ideally employ a multidisciplinary team of physicians including medical oncologists, urologists, and radiation oncologists at the respective institutions, rather than single physician practices in a private setting. Several large academic centers have instituted multi‐disciplinary guidelines for following patients on personalized AS protocols, similar to those proposed by D'Amico et al. 22. including a risk‐based assessment scheme that may also incorporate comorbidity, ethnicity, family history, MRI imaging characteristics, and even genomic testing.

Also, this study revealed significant preferential use of AS in uninsured patients and the overutilization of curative intervention in those who were privately insured. These nonclinical factors are important to recognize given the rising cost of modern healthcare. Several groups have formally studied the impact of AS compared to immediate treatment for prostate cancer on health care costs 23, 24. Recent data by Laviana et al. 25 examining the time driven activity‐based cost for competing treatments of low‐risk prostate cancer suggests that cost can range from $7298 for AS (including transrectal ultrasound biopsy and multiparametric MRI imaging) to $23 565 for IMRT, and even more for proton therapy or RP 26. Even with long‐term follow‐up, AS results in a cost benefit of 35% compared to initial treatment for localized prostate cancer, resulting in an estimated $1.9 billion dollar savings 24. Thus, in the existing fee‐for‐service model, the significant cost “savings” for patients undergoing AS may be undesired by financial institutions, but appropriate management of those eligible for AS is a major public health concern and significantly impacts health care costs.

In addition to preconceived notions by providers with regards to outcomes based on age, ethnicity, or even financial outcomes for their institutions, there may be additional factors that lead to recommendations and treatments other than AS. This includes findings on multiparametric MRI imaging, which unfortunately are not well characterized in this dataset. It has been demonstrated that occult higher grade malignancy or extraprostatic disease [excluding a patient from AS] may be found more effectively using diffusion‐weight MRI sequences within the PIRADS (prostate imagine reporting and data system) classification 27. Certainly, MRI–ultrasound fusion biopsies may improve the detection of higher grade prostate cancer, particularly in the anterior and peripheral zones 28, and this has been incorporated into the most recent NCCN guidelines 21 as well as institutional AS programs (NCT0858688).

With respect to patient preference, although repeat prostate biopsies may not be indicated for those with a life expectancy of less than 10 years, many patients may be reluctant to undergo routine annual biopsies. In addition, what is not well characterized within the clinical literature is the potential raised level of cancer‐related anxiety of patients who undergo AS. A study by Hayes et al. utilized a decision analysis simulation model and revealed that AS is associated with the highest quality‐adjusted life expectancy when compared to active intervention, including RT or RP 29. Bellardita et al. 30. found that a major component of the psychological burden of patients (within the first year of follow‐up) undergoing AS was potentially the lack of physician support for conservative management as well as social network of support favoring more aggressive intervention.

As one of the most modern studies prospectively analyzing AS, the ProtecT (Prostate Testing for Cancer and Treatment) study is the first study of PSA‐screened men with favorable‐risk prostate cancer randomized to AS versus treatment (RP or RT), and was designed to stratify by age, T‐stage, PSA, and GS, without controlling for race, PSA density, or co‐morbidity 19. Also, contrary to our current study, the ProtecT trial may not truly reflect the general US population as the age limit in their study was 69 years, and <1% of patients were of African‐Caribbean ethnicity 19, 31.

Nevertheless, there are several limitations to this study. One such limitation of this particular dataset involves selection bias and representation of hospital‐based registries that contribute cases annually to the NCDB. This may limit evaluation of patients receiving care at outpatient community centers that are not accredited by the ACS/CoC. Second, although this is a contemporary dataset, our study may not reflect nationwide patterns that are current in 2016 and beyond. We postulate that this may increase in popularity in the coming years, particularly with additional prospective studies demonstrating excellent outcomes with AS. Third, the NCDB does not report PSA density (serum PSA/Prostate volume) which may be superior to serum PSA and GS alone for adverse pathologic features prediction in patients with clinically localized prostate cancer 32. Fourth, there are unobserved variables in this dataset that may be incorporated in modern day practice to better predict patients who are likely to harbor more aggressive disease, that is, genomic tests 33, 34. Lastly, as discussed above, the NCDB does not code information regarding the use of, and findings from, multiparametric MRI studies that may have been performed and led to decisions to pursue curative intervention(s), rather than AS.

We should note that the current nationwide study has several advantages and appears to complement and validate the existing institutional literature on AS. For example, in contrast to SEER‐Medicare studies that investigate billing code‐derived events, predominately for patients 65 or older [for whom AS may be commonly chosen regardless], our study was based on a prospectively collected registry that includes patients under 65 years of age, which allows the inclusion of those with a life expectancy of >20 years. In addition, all of the currently published studies examining AS accumulate to less than 3000 patients in total, predominately from single‐institution experiences, whereas our cohort represents a variety of institutions and patient mix, and is the largest single study examining disparities in its utilization. In fact, our study may be most representative of the entire US population–minority patients comprised 13% of the cohort, whereas one of largest previously reported studies only included up to 7.4% black patients 13. In addition, we have utilized a generalized estimating equations approach to account for subject‐within‐cluster (facility) correlations that may reflect provider/institutional practice patterns.

Given the multiple clinical and nonclinical factors that appear to determine the patient's road to AS or active intervention, we suggest that multidisciplinary teams should continue to offer a comprehensive and accurate clinical program incorporating AS that abides by national guidelines and is reassuring to their patients. In addition to the growing body of evidence of the safety of AS 31, efforts such as the recent ASCO endorsement of Cancer Care Ontario Guidelines may increase awareness in communities across the nation, particularly where AS may be prescribed the least 20. The current low, but rising rates of AS and the apparent disparities in its prescription, may be an opportunity for the medical community to improve the quality of life of our patients [by avoiding harm from unnecessary treatment] while subsequently reducing the financial burden on the healthcare system.

## Conflicts of Interest

There are no conflicts of interest disclosures from any authors.
